# The Potential of Nano-Based Photodynamic Treatment as a Therapy against Oral Leukoplakia: A Narrative Review

**DOI:** 10.3390/jcm12216819

**Published:** 2023-10-28

**Authors:** Angela Angjelova, Elena Jovanova, Alessandro Polizzi, Simona Santonocito, Antonino Lo Giudice, Gaetano Isola

**Affiliations:** 1 University Dental Clinical Center St. Pantelejmon, Skopje, Faculty of Dentistry, Ss. Cyril and Methodius University in Skopje, 1000 Skopje, North Macedonia; angela.angjelova@students.stomfak.ukim.mk (A.A.); elena.jovanova@students.stomfak.ukim.mk (E.J.); 2 Department of General Surgery and Surgical-Medical Specialties, School of Dentistry, University of Catania, 95124 Catania, Italy; simona.santonocito@phd.unict.it (S.S.); antonino.logiudice@unict.it (A.L.G.); gaetano.isola@unict.it (G.I.)

**Keywords:** nanoparticles, photodynamic therapy, oral leukoplakia, nanotechnology, nano-based

## Abstract

Oral leukoplakia is a predominantly white lesion of the oral mucosa that cannot be classified as any other definable lesion with the risk of progressing into malignancy. Despite the advancements in conventional therapy, the rates of malignant transformation remain notably high, affecting 4.11% of adults, due to the difficulty of accurate diagnosis and indistinct treatment. Photodynamic therapy (PDT), being a minimally invasive surgical intervention, employs a variety of factors, including light, nano-photosensitizers (PSs) and oxygen in the management of precancerous lesions. PDT faces limitations in administering photosensitizers (PSs) because of their low water solubility. However, these challenges could be effectively resolved through the incorporation of PSs in nanostructured drug delivery systems, such as gold nanoparticles, micelles, liposomes, metal nanoparticles, dendrimers and quantum dots. This review will give an overview of the different innovative PS approaches in the management of premalignant lesions, highlighting the most recent advancements. From a clinical perspective, it is expected that nanotechnology will overcome barriers faced by traditional therapeutics and will address critical gaps in clinical cancer care.

## 1. Introduction

The typical precursor to potential malignancy that may arise from oral squamous cell carcinoma (OSCC) is considered to be oral leukoplakia (OL) [1,2]. Thereby, the WHO Collaborating Centre defined it as “A predominantly white plaque of questionable risk having excluded (other) known diseases or disorders that carry no increased risk of cancer” [3]. A global occurrence estimated by a published meta-analysis revealed that 4.11% of adults experienced OL; conversely, the progression to malignancy shows a fluctuation extent from 7.7% to 38.1% [2]. Leukoplakia is typically diagnosed following the onset of the fourth decade of life, and they exhibit a higher prevalence in males and smokers [4]. There is no association of chemical or physical causes with oral leukoplakia other than smoking, established in 1994 after an international symposium held in Uppsala, Sweden [1]. However, some studies show that tobacco, alcohol, dentures that do not fit, mechanical injuries, infections caused by *Candida albicans*, and some viruses (EBV, HPV, HSV, HIV) are considered to be additional factors that significantly increase the development of OL [4].

Photodynamic therapy (PDT), as a potential noninvasive therapeutic tool, presents a new and alternative, non-surgical treatment that uses photosensitizing agents accumulating selectively in the tissues activated by exposure to a light source at a specific wavelength. This method involves light source, photosensitizers and tissue oxygen resulting in oxidative cell death and damage by the process of necrosis, apoptosis or autophagy [5]. PDT consists of different phases and characteristics:

1. Photosensitizer Administration: A photosensitizing drug is either applied topically to the skin or administered intravenously, depending on the condition being treated. This drug is designed to accumulate in the target cells or tissue.
2. Waiting Period: After the photosensitizer is administered, a waiting period is required. This allows time for the drug to be absorbed by the target cells while clearing from the surrounding healthy tissue.
3. Light Activation: A specific wavelength of light, often delivered through a laser or non-thermal light source, is directed at the area of interest. This light activates the photosensitizer that accumulates in the target cells.
4. Photochemical Reaction: When the photosensitizer is exposed to activating light, it reacts with oxygen, producing a form of oxygen called singlet oxygen. This highly reactive oxygen species damages and destroys the target cells, leading to their death.
5. Selective Treatment: PDT is designed to be selective, targeting primarily the cells that have absorbed the photosensitizer, sparing surrounding healthy tissue [6].

Photosensitizers (PS) have two concurrent reactions: Type I electron transfer reactions occur characterized by the direct interaction of PS with cellular elements, resulting in the formation of either anionic or cationic radicals. These radicals subsequently engage with molecular oxygen, resulting in the generation of reactive oxygen species (ROS). Second, Type II energy transfer reactions take place, wherein the PS directly reacts with molecular oxygen, yielding singlet oxygen [7].

Various studies have investigated the potential anti-tumor effect of PDT, exploring mechanisms that involve the direct infliction of destruction of tumor cells, impairment of the vasculature and the initiation of inflammatory and immune responses [8]. The application of PDT is widely used and rapidly growing in the treatment of precancerous lesions, particularly within the oral cavity and has been used for the management of potentially malignant disorders such as OL [9].

**Figure 1:**
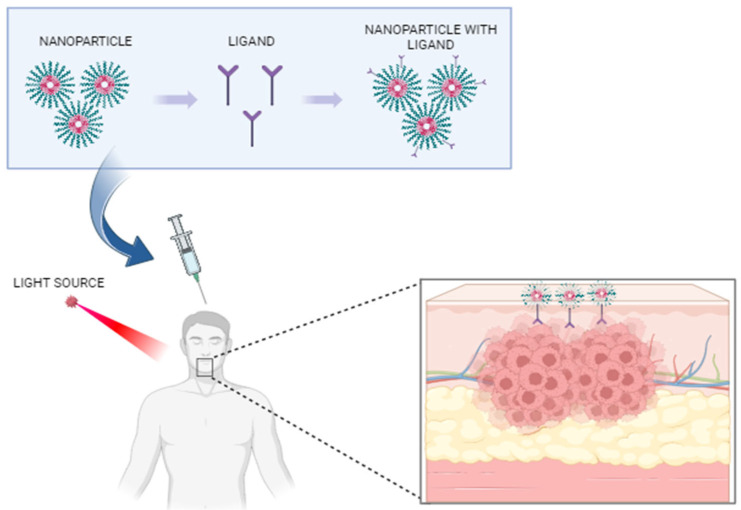
Application of nanotechnology for in vivo bioimaging of oral premalignant lesions. Created with BioRender.com, accessed on 20 September 2023.

There has been a significant focus in recent years on the exploration of nanomaterial-based PDT (Figure 1). This emerging therapeutic approach utilizes nanomaterials either as carriers or photosensitizers (PS), opening new avenues for treatment [10]. Nanomaterials refer to particles with a sub-micrometer size that can selectively target the tumor cells [11].

They possess distinctive characteristics that are more unique than organic photosensitizers because they are more stable under irradiation and have good optical properties that result in enhanced penetration and effectiveness of the PDT [12]. Moreover, nanomaterials are used as a carrier for photosensitizers, which enables the delivery of the same approaching the tumor as a result of the large surface zone and their adaptable surface [12]. Besides protecting the drug and reducing side effects, nanoparticles with mucoadhesive and mucopenetrating characteristics can extend the duration of contact between the formulation and oral mucosal tissues, thereby enhancing drug delivery to subepithelial cells [13].

Since conventional therapeutic strategies have considerable drawbacks and adverse consequences in clinical applications, there has been a requirement for effective treatment for OL. Nanotechnology and numerous forms of nanoparticles are unique fields because of their customizable surface modification and their ability to achieve immediate and full biocompatibility. It has the capacity to change the way healthcare is delivered, moving from the treatment of diseases in broad populations to providing individualized and precise treatments for each patient [14]. Moreover, nanotechnology can be employed to ensure patient safety by preventing overdosing and the spontaneous degradation of orally administered drugs [15].

This article will highlight the approach in the field of nanotechnology and its impact on treating premalignant lesions. Moreover, different types of nanoparticles that have been developed for drug delivery in the therapy of oral leukoplakia will be covered in this study. More specifically, the properties of gold nanoparticles, micelles, liposomes, metal nanoparticles, dendrimers and quantum dots that have successfully increased the success in the treatment of cancer cells will be explored.

## 2. Oral Leukoplakia

Leukoplakias have been conventionally classified into two distinct forms based on clinical characteristics [16]. Homogenous type is described as a lesion that appears uniformly white and the surface exhibits either a flat or slightly wrinkled texture [16]. The other form is referred to as non-homogenous. This type has three distinct clinical variations and typically exhibits symptoms:

1. Speckled—characterized by a mixed white and red appearance (also referred to as erythroleukoplakia) with white attributes predominating [4].
2. Nodular—featuring small polypoid outgrowths presenting as rounded red or white protuberances [4].
3. Verrucous or exophytic—displaying a surface appearance that is either wrinkled or corrugated [4].

In instances where OL spreads extensively throughout the oral cavity, the condition is termed proliferative verrucous leukoplakia (PVL) [17]. Frequently, instances of oral white patches arise as a result of discernible localized irritation; for instance, thickened hyperkeratotic alterations are commonly observed in regions lacking teeth, specifically on the alveolar ridges. This is particularly notable among individuals who do not utilize a dental prosthesis in these areas [18].

### 2.1. Diagnosis of Oral Leukoplakia

Leukoplakia has the potential to manifest in any region within the oral cavity, mostly situated on the floor of the mouth, soft palate and the ventral surface of the tongue (sublingual keratosis), while when found in other regions, they may be classified as having lower risk of malignancy [16]. Frequently, leukoplakia lacks noticeable symptoms in other instance [16]. During the progression of the lesion, the individual might become aware of an ulcer that does not heal. Subsequent stages manifest with symptoms such as bleeding, tooth mobility, changes with denture use, dysphagia, dysarthria, odynophagia and the emergence of a mass in the neck [18].

Clinical assessment relies predominantly on tactile examination and visual observation [16]. A preliminary clinical diagnosis of leukoplakia is established in the presence of a white patch after ruling out a local traumatic origin. This is confirmed when a patch cannot be removed through scraping and when its color remains unchanged upon tissue stretching. Additionally, careful attention should be paid to excluding other conditions that manifest as white in color during clinical examination [4]. Unfortunately, progress in the early detection of lesions is constrained, which is largely attributable to a substantial number of patients pursuing diagnosis and intervention only when their condition has reached Stage III and Stage IV [18]. However, OL is a diagnosis reached through the process of elimination. All established origins of white oral lesions must be excluded prior to arriving at this clinical diagnosis. Before performing a biopsy, it is essential to rule out any potential contributing factors within a duration of 2 to 4 weeks. Should the lesion persist beyond this period, a biopsy is recommended to establish a diagnosis and assess the potential for malignancy. If redness, erosion, ulceration or induration is evident, the biopsy site should correspond to these characteristics and in the cases of extensive or multifocal lesions, multiple biopsies might be required [19]. As a general guideline, each instance of leukoplakia should undergo biopsy, regardless of symptom presence, clinical subtype, size or location within the oral cavity [16].

### 2.2. Prognosis of Oral Leukoplakia

Studies reveal that a relatively small percentage, ranging from less than 1% to 18%, of oral premalignant lesions progress to oral cancer. Furthermore, specific clinical subtypes of leukoplakia have been identified as having a heightened susceptibility to undergoing malignant transformation. Notably, epithelial neoplasia may hold greater significance in the prognosis of progressing into malignancy compared to clinical attributes [20].

Several predictive elements for malignant transformation that occur are lesion size, the clinical subtype, oral location and presence or absence of epithelial dysplasia. However, when applied to individual patients, these factors are devoid of consistent reliability [16]. Nevertheless, there remains a restricted comprehension of the future outlook for carcinomas originating from proliferative verrucous leukoplakia [21]. Additionally, there is supportive research regarding the exploration of dysplastic characteristics proximate to carcinomas [20]. One study examines the occurrence and dispersion of indicators like keratins, p53, epidermal growth factor receptor and chromosome instability. These indicators may have potential associations with the carcinogenic process and the concept of field cancerization [20]. Contemporary theories regarding tumorigenesis underscore the presence of molecularly modified paraneoplastic domains, giving rise to the potential development of multiple lesions. Furthermore, it has been demonstrated that extensive lesions exhibit elevated rates of malignant transformation in comparison to their more localized counterparts [22].

## 3. Principles of Nanotechnology

The benefits of utilizing nano-based photodynamic therapy contribute to addressing the associated obstacle; therefore, the innovative domain, nanotechnology, has brought a transformative impact on the industry itself and it possesses the potential to enhance diagnostic accuracy using less agents that can have a negative impact on the tissue, while also establishing contemporary treatment protocols [23]. There has been a continuous expansion in the field of nanotechnology, as the advantages become increasingly evident [24]. This incorporation of nanotechnology in early detection, treatment and patient management significantly elevates the clinical methodology [23].

Today, nanotechnologies have the aspiration of enhancing pharmacokinetics and pharmacodynamics (PK/PD), therapeutic effectiveness and selectivity that will exhibit particular preference for malignant cells [24]. Precisely directed release of the drug can reduce the necessary dosage to a minimum due to the inherent attraction and targeted binding facilitated by ligands, consequently leading to diminished overall toxicity and thereby enhancing both effectiveness and the quality of life for patients [24].

Table 1 resumes the beneficial impact of nanomedicines on potentially malignant lesions.

### 3.1. Nanoparticles

Densely packed supramolecular formations that possess dimensions ranging between 10 and 1000 μm are referred to as nanoparticles (NPs) [23]. Measured less than 10 nm can be readily eliminated by renal filtration, whereas particles exceeding 200 nm can be phagocyted within the reticuloendothelial system (RES). Smaller than 100 nm tends to have prolonged circulation in the blood flow [25]. Consequently, nanoparticles appear to align with the optimal size for manipulation purposes. Nanoparticles could potentially demonstrate attributes such as selectivity, pH-sensibility, self-organization, constancy, pharmaceutical encapsulation and tissue compatibility [23]. The pH responsive nanoparticle systems are formulated to maintain stability at a physiological pH of 7.4; however, they undergo deterioration to liberate the medication within specific tissues that possess lower pH levels than the physiological range, as exemplified by the acidic conditions of neoplastic cells [26]. They have diverse medical applications for both diagnostics and therapeutics, serving as a delivery platform enabled by their capacity to encapsulate pharmacological or genetic materials and effectively enter cells within specific organs [27].

**Figure 2:**
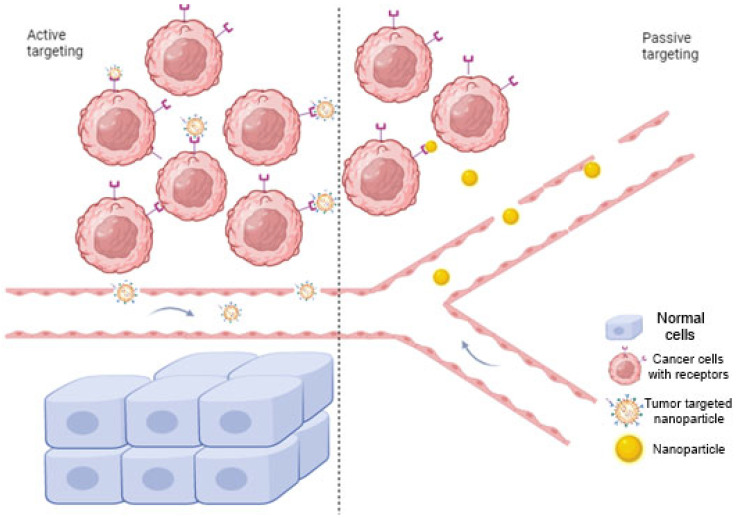
Mechanism of nanoparticle drug delivery via two main mechanisms: active and passive targeting. In active targeting, specific ligands bind to the receptors on the tumor cells, while passive targeting involves nanocarriers that pass through leaky walls and accumulate at the tumor site due to the enhanced EPR effect. Created with BioRender.com, accessed on 20 September 2023.

NPs can facilitate the transportation of the medication to the desired tissue through two main mechanisms: passive tumor targeting (so-called permeability and retention effect- EPR) and targeting based on a specific ligand interaction called active targeting [26] (Figure 2). Nanoparticles for solid tumors have the capability for targeted delivery, relying on the permeability and retention (EPR) effect, a result of abnormal neovascularization-induced leakage in tumors that leads to preferential accumulation of nanoparticles in tumor regions due to the integrity of blood vessels in normal tissues, enabling positive targeting delivery without ligands [28]. Ligand-mediated targeting involves attaching ligands to the outer surface of the NPs and forming connections with suitable receptors situated at the cancerous cells. The ligands fall into categories such as proteins, nucleic acids or other types that exhibit affinity for receptors that are specifically abundant neoplastic cells or the circulatory system [26].

In the context of disease treatment, targeted therapy refers to the strategy of transporting precise quantities of therapeutic agents to the affected anatomical site over an extended timeframe. To realize this objective, the advancement of safer and more efficient therapeutic nanoparticles is imperative, representing a fundamental objective within the field of nanomedicine [25].

### 3.2. Particle’s Shape

Characteristics such as the surface, charge and size of nanoparticles significantly influence drug delivery systems. One of the primary factors guiding interactions and the nano-bio interface is the surface charge of nanoparticles. The capacity of nanomaterials to enter cells relies on their surface charge. It substantially influences the process of cellular endocytosis and in a general context, nanomaterials with a positive charge might undergo efficient uptake by cell membranes due to the negatively charged character of the cellular surface [29]. As a result, in order to extend the duration of nanocarrier circulation within the bloodstream and to ensure the effective delivery of drugs to specific targets, the reduction of opsonization emerges as a crucial determinant. This potential reduction in opsonization can be potentially achieved through the application of surface coating on carriers utilizing hydrophilic substances like polyethylene glycol (PEG) [30].

The form of nanoparticles also affects their exposure, rate and distribution in drug delivery [31]. Most nanocarriers are synthesized in a spherical configuration primarily designed to deliver anticancer agents. In contrast, viral and bacterial nanocarriers exhibit diverse geometries, such as filaments and cylinders [30]. Consequently, nanoparticle shape impacts macrophage uptake and subsequent clearance. The alignment of nanoparticles during interaction with macrophages is influenced by their configuration, which is closely tied to the manufacturing process for creating non-spherical particles [31]. Nanostructures consisting of nanoparticle cores surrounded by a ligand shell exhibit distinct interactions with cell membranes due to variations in the way surface ligands are presented [32].

While many NPs typically exhibit a spherical shape, advancements in nanofabrication have led to the creation of diverse NP shapes with unique geometric physical and chemical attributes. For instance, innovative contrast agents for molecular imaging and photo thermal cancer treatment have been developed using nanorods [33]. As an example, elongated cationic nanoparticles demonstrate a greater propensity for endosomal uptake in comparison to cationic nanoparticles with different geometries. This phenomenon indicates that immune system cells could perceive these nanoparticles in a manner similar to how they recognize rod-shaped bacteria [25]. Additionally, asymmetrical functionalization of gold NPs has facilitated the assembly of nanochains [33].

### 3.3. Range of Nanoparticles

**Figure 3:**
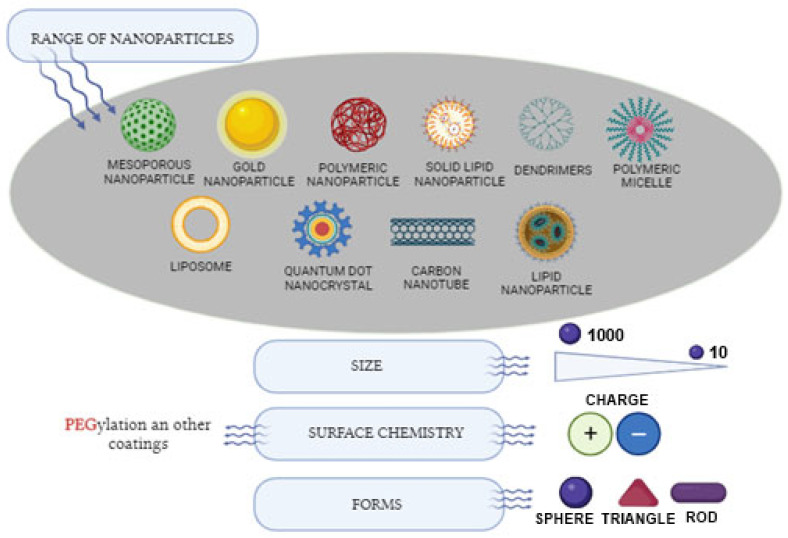
Range of nanoparticles utilized in therapeutic applications. Created with BioRender.com, accessed on 20 September 2023.

A range of nanomaterials (NMs) have received clinical approval and numerous others are presently undergoing clinical testing (Figure 3). These diagnoses and therapeutic NMs can be typically categorized into two groups:

1. Organic NMs encompassing liposomes, polymers, micelles;
2. Inorganic NMs include substances like metal nanoparticles and metal oxides, carbon-based materials and mesoporous silica nanoparticles [29].

There are also two other primary classifications: nano-structured and nanocrystalline. The subset of nano-structured materials can be delineated into polymer-based, non-polymeric and lipid-based nanoparticles.

Non-polymeric NPs consist of carbon nanotubes, nanodiamonds, metallic nanoparticles, quantum dots and nanoparticles that are based on nano-silica.

Lipid-based NPs are further categorized into two subdivisions: liposomes and solid lipid NPs [25].

#### 3.3.1. Inorganic Nanoparticles

This classification of nanomaterials constitutes a notable portion of contemporary drug transport mechanisms, attributed to their meticulous regulation of dimensions and configuration, adjustable physicochemical attributes, regulated surface chemistry and versatile multifunctionality [29].

##### Metal Nanoparticle and Metal Oxides

Among various types of nanoparticles, metallic NPs offer notable benefits, including excellent biocompatibility and stability, modifiable size, favorable optical characteristics, effortless surface customization and prolonged functional activity.

Metal oxide nanomaterials have demonstrated potential in biomedical applications for fluorescent labeling owing to their favorable attributes, such as exceptional photostability, substantial extinction coefficient, elevated emission quantum yield and straightforward surface customization. These nanoparticles can serve as photosensitizers, carriers for drug delivery and up-conversion systems, thereby increasing the transport of chemotherapy agents, radionuclides and antibody drug cancerous cells [34].

Gold nanoparticles exhibit notable chemical stability and strong compatibility with biological systems. These attributes make them sufficient agents for passivization, carriers for drug delivery and manifest diverse geometries such as particle rod, cluster, shell, spike and star. Within PDT, gold nanoparticles find utility both independently and with versatile nanomaterial hybrids to facilitate the delivery of PSs for therapeutical purpose [34]. A nanoparticle delivery mechanism was formulated comprising TNF-alpha loaded PEG coated gold NPs designed to enhance tumor destruction while mitigating the systemic toxicity associated with TNF-alpha [35].

Silver nanoparticles are found in delivering drugs capable of enhancing the therapeutic effectiveness of medications. These particles, when combined with phytopharmaceuticals, can serve multiple roles in cancer therapy, such as safe delivery carriers, contrast agents, and photo thermal agents. Compared to gold NPs and Pt NPs, silver nanoparticles exhibit higher efficiency in generating singlet oxygen. Furthermore, they have displayed the ability to extend the persistence of immune memory effects for a duration of 40 days [29].

Iron oxide nanoparticles (IONPs) have introduced a diverse array of promising biomedicine applications owing to the presence of multiple functionalities within a single nanostructure. IONPs function as contrast agents in both magnetic resonance imaging (MRI) and magnetic particle imaging (MPI) that enable the non-invasive visualization of inflammation processes and facilitate the monitoring of therapeutic cell [36]. They are utilized for the transportation of ovalbumin (OVA), and the integration of iron oxide nanoparticles with OVA substantially enhances the activation of immune cells and the synthesis of cytokines, resulting in robust humoral and cellular immune reactions. The evidence provided that these nanoparticles significantly suppressed tumor spread in experimental mice while maintaining tissue compatibility [29].

##### Carbon-Based Materials

Considerable research has been conducted on carbon-based nanomaterials for purposes of cancer imaging, delivery and diagnosis. This is attributed to their appealing attributes, which encompass substantial surface area, considerable drug loading capability and surfaces that can be readily modified. They also display antimicrobial efficacy and it has been suggested that these nanomaterials induce oxidative stress, resulting in bacterial membrane impairment. However, recent research has indicated that the fundamental antimicrobial mechanism of carbon-based nanomaterials is primarily attributed to their physical interaction with bacteria rather than oxidative stress [29].

Carbon nanotubes are regarded as viable transporters in pharmaceutical transportation due to their attributes such as ordered arrangement, exceptionally low mass, elevated electrical and thermal conductance and their substantial top surface area. They represent carbon-based nanomaterials with reduced dimensions, capable of facilitating the conveyance of diverse therapeutic agents, including anti-cancer and anti-inflammatory substances. Nevertheless, the non-solubility of carbon nanotubes (CNTs) can give rise to health challenges; for instance, non-functionalized CNTs have the potential to gather in pulmonary tissue, resulting in pulmonary inflammation and toxicity [37]. Additionally, they have found utility in fabricating biosensors for identifying generic irregularities and molecular anomalies, as well as in drug delivery mechanisms, encompassing diverse agents for detection and treatment purposes [30]. Recent inquiries into multi-walled carbon nanotubes (MWCNTs) for the concurrent delivery of drugs have demonstrated that controlled drug release at the cancer site and cellular uptake hold promise in the treatment of multi-drug-resistant cancer [29].

##### Mesoporous Silica-Based Nanomaterials

Silica materials (MSNs) have surfaced as an alternative category of pharmaceutical transport systems, owing to attributes like their expansive surface area, consistent porosity, durability, minimal toxicity and tightly constrained size distribution [29]. Multiple scientists have attempted to enclose NPs within silica nanoparticles with the aim of facilitating their targeted delivery into cancer cells [38]. The defining trait of mesoporous silica is marked by the existence of mesopores, affording the material an extensive surface area [39]. Mesoporous silica nanoparticles are fabricated through a sol-gel process for the creation of nanoparticles with consistent sizes and the inclusion of a surfactant during this synthesis leads to the formation of a configuration characterized by numerous tiny pores, thereby proceeding to a mesoporous structure [40].

#### 3.3.2. Organic Nanoparticles

##### Liposomes

Liposomes were the initial focus of exploration as pharmaceutical conveyors. These carriers exist on a nano-scale and possess a colloidal nature, typically exhibiting dimensions spanning 80–300 nm, consisting of globular vesicles made up of manufactured or organic phospholipids and steroidal compounds such as cholesterol and they form bilayers or alternative surface-active agents that undergo automatic formation if specific lipid varieties disperse within aqueous environments [41]. These nanoparticles are favorable transport systems for intelligent conveyance of compounds, such as hydrophilic or hydrophobic attributes. They offer multiple benefits, including a membrane structure resembling cells, biocompatibility, minimal immunogenicity as well as improvements in safety and therapeutic effectiveness [42]. They exhibit remarkable efficacy in facilitating the diffusion of their delivery through the plasma membrane stem from their distinctive composition and structural arrangement [43]. Because of their fast degradation and brief duration of presence in the bloodstream, the usual liposomes demonstrate limited potency in achieving elevated ratios of tumor accumulation compared to normal tissue. Research efforts have focused on surface modifications to generate what are known as long-circulating liposomes characterized by improved stability in the bloodstream [8].

In contrast to polymer nanoparticles, these liposomes have reduced stability and pose greater challenges in managing their controlled release characteristics [43].

##### Polymeric Nanoparticles

Polymeric nanoparticles are colloidal nanoscale particles in which therapeutic molecules are enclosed, adsorbed or linked within the polymer matrix [29]. They can systematically release medicinal compounds via controlled mechanisms, such as surface erosion of NPs, drug diffusion of the polymer matrix or expansion followed by diffusion [44].

Polymeric nanoparticles (PNPs) exhibit dimensions spanning 10 to 100 nm. In accordance with their observed behavior within living organisms, PNPs can be categorized into biodegradable variants and non-biodegradable forms [41]. They have captured attention within utilization for therapeutic purposes due to their adaptability of structure derived from functionalization capabilities, methods of synthesizing large molecules and the variety of available polymers [45].

Polymer-based NPs contain a range of structures, such as dendrimers, micelles, nanogels, protein nanoparticles and drug conjugates [25].

##### Micelles

Polymeric micelles are self-aggregating nanoscale formations consisting of a central hydrophobic core that envelopes medications with limited solubility in water. These micelles possess a hydrophilic outer layer serving the dual purposes of shielding the drug within an aqueous setting and strengthening the micelles’ stability in opposition to the detection within a living organism through the RES [46]. Additional fundamental traits of polymeric micelles comprise their minor size ranging from 10 to 100 nm, which facilitates their buildup in tumor cells, their capacity for non-specific relation with organic constituents due to the presence of biologic polymer capsules, their ability to control the liberation of medication and their potential for altering the composition to enhance their attributes [46,47].

#### 3.3.3. Dendrimers

Dendrimers represent a category of extensively three-dimensional branched synthetic polymers that arrange into spherical macromolecular structures [46]. These nanoscale polymers displayed exceptional biological traits, encompassing diminutive dimensions, water solubility and uniform structure [46]. Furthermore, owing to the presence of active moieties on their subdivisions, they are adept at accommodating and associating with a variety of hydrophilic and hydrophobic medical compounds. They have been utilized in the transport of anti-cancer agents, where the pharmaceutical substances are either encapsulated within or linked to dendrimers [29]. Dendrimers can be categorized into separate domains: the central core, the internal branches and the outer comprising surface groups [48]. Several instances of nanoscale molecules exhibiting dendritic architecture incorporate glycogen, amylopectin and proteoglycans [41].

#### 3.3.4. Nanogels

Nanogels consist of hydrophilic polymers combined with water, functioning as carriers for therapeutic substances that display enhanced efficacy compared to alternative nanocarriers due to their substantial water content, notable porosity, remarkable ability to accommodate both hydrophobic and hydrophilic medications, strong biodegradability and compatibility with diverse bioactive agents such as nucleic acids and proteins. Additionally, nanogels demonstrate heightened reliability and extended circulation within the bloodstream, culminating in an augmentation of the medication in neoplasms [49,50]. Following exposure to photo irradiation, the nanogels comprised of these light-responsive polymers will experience specific phase alterations driven by modifications in the structure or polarity of their chemical moieties with specific roles [51].

#### 3.3.5. Quantum Dots

Quantum dots, alternatively referred to as semiconductors or nanocrystals, possess modifiable nanoscopic characteristics within a size spectrum spanning 100 to 200 nm. These entities are molded into diverse configurations and enveloped with various biomaterials, whereas under ultaraviolet illumination, these dots display luminescence [52]. The central aim revolves around fabricating diminutive probes capable of infiltrating cellular structures and organelles, while simultaneously exhibiting notable specificity, versatility and durability [53]. Targeting cancerous tissue for treatment purposes has predominantly concentrated on a limited selection of potential ligands. These ligands correspond to receptors that are characterized by their heightened expression within tumor cells. Notably, folic acid stands as an exemplary ligand in this context, with extensive utilization as a targeting molecule for the delivery of treatment agents for cancerous cells. This preference arises from folic acid’s remarkable capacity to strongly bind with the folate receptor, thereby facilitating targeted drug delivery [54].

Furthermore, they possess potential utility within PDT as photosensitizing agents capable of facilitating precise cellular ablation, exhibit the capacity to attach to antibodies situated on the outer part of targeted cells upon activation by ultraviolet irradiation and liberate a reactive oxygen species (ROS), resulting in a destruction of the targeted cells [55]. They have the potential not solely limited to their utilization as probes or conveyors for pharmaceutical transport within cancer treatment, but also extend to generating thermal energy upon irradiation [56]. The optimal nanocarrier materials should demonstrate the subsequent attributes: absence of reactivity with pharmaceutical compounds, efficient encapsulation, suitable techniques for purification and production, favorable biocompatibility and minimal toxic effect, extended in vivo retention period, etc. [54].

Table 2 and Table 3 summarize the advantages and disadvantages of nanoparticles for oral leukoplakia management and the main drawbacks related to their utilization.

## 4. The Impact and Future Perspectives of Nanotechnology on Oral Leukoplakia

In an ideal scenario for medicines to achieve effectiveness in therapy, they have to be capable of efficiently traversing the tissue with less reduction of volume within the bloodstream to access the targeted cells. These drugs should exhibit the capacity to specifically eliminate dysplastic cells while sparing healthy ones [57]. A variety of nanomaterials have already been searched and obtained clinical approval, while numerous others are presently in the process of clinical evaluation, for instance: liposomes, dendrimers, polymeric NPs, quantum dots, as well as materials such as iron oxide, gold NPs and silica-based NPs [23,58].

Within quantum dots, in scientific terms, light can be employed to generate oxygen radicals with elevated levels of energetic power, which can undergo chemical reactions leading to the destruction of adjacent organic molecules, such as those found in cancerous cells. It differs from chemotherapy in that it does not disseminate reactive molecules throughout the body, thereby avoiding toxicity; instead, it exclusively targets areas where light is applied and these oxygen atoms are present [59].

El-Hamid and colleagues conducted a study to examine the apoptotic impact of DOX and PEGylated liposomal doxorubicin (PLD) on Cal-27 cells. Concurrently, Narayanan and co-researchers encapsulated a liposomal nanocarrier and assessed its anticancer effectiveness in squamous tumor cells. Their findings demonstrated that the mixture of medicines enclosed within the liposomal layer exhibited superior efficacy when compared to administering the drugs independently [60].

Precise drug delivery mechanisms can lead to a reduction in the necessary dosage, subsequently diminishing the general perniciousness level. This enhancement contributes to heightened effectiveness and improved wellbeing [24]. The utilization of nanotechnology-drive drug delivery approaches holds promise in enhancing the therapeutic efficacy for dysplastic cells. This offers advantages such as bioeffectiveness, customized allocation and limited adverse effects [23].

Unipolar research indicates that PDT could serve as a viable therapeutic choice for oral leukoplakia, especially in instances of absent, mild or moderate dysplasia. Nonetheless, several variables can influence the treatment, encompassing factors such as the light source, wavelength and duration of application. Favorable outcomes are observed when a laser engaged as a light source has a wavelength that goes beyond 640 nm [61].

The implementation of nanotechnology in the field of precancerous lesion management has undergone rapid and significant expansion, as evidenced by the increasing volume of research studies encompassing both nanomedicine and technology [62]. Consequently, innovative therapeutic agents offer the potential to selectively eradicate dysplastic cells while preserving healthy ones, thereby potentially leading to heightened survivability [23]. Indeed, the latest progress in research-based science is making it progressively more possible to develop specialized or multifaceted nanotechnology outcomes tailored for medical purposes by precisely targeting the affected cells or tissues and minimizing the side effects and they can offer significant advances over non-targeted drug delivery methods (Table 4). However, successful implementation will require careful consideration of factors such as safety, efficacy and regulatory approvals [63,64].

In the context of premalignant lesion therapy, the emerging frontier is nanotechnology, which holds significant promise for enhancing OL treatment outcomes. This approach offers potential advancements on dual fronts: firstly, by imparting novel attributes to pharmaceutical agents, such as enhanced stability, altered pharmacokinetics, and reduced toxicity; and secondly, by directing these agents specifically towards tumor sites. One prospective strategy involves the combination of anti-tumor drugs with nanoparticles, with the objective of surmounting both noncellular and cellular resistance mechanisms while heightening drug selectivity for dysplastic cells and concurrently diminishing their toxicity to healthy tissues [65].

## 5. Conclusions

Utilizing nanoparticles within the photodynamic drug delivery system has become a topic of significant interest and holds substantial promise for its application in oral leukoplakia treatment. The new frontier in potentially malignant oral lesions therapy is nanotechnology, which has enormous potential for improving the results of treatment. This strategy provides potential improvements by giving pharmaceutical drugs additional properties, including improved stability, changed pharmacokinetics, and decreased toxicity. Moreover, nanoparticles can target these substances directly at tumor locations. One potential approach included combining anti-tumor medications with nanoparticles in order to overcome both cellular and noncellular resistance mechanisms, increasing treatment selectivity for dysplastic cells, and simultaneously reducing drug toxicity to healthy tissues. Notwithstanding the benefits of nano-based therapy, the transfer of innovations to medical practice remains a complex and demanding assignment that requires further exploration. However, they are expected as potential next-generation nanomedicines capable of significantly improving premalignant management strategies. Overall, nanotechnology holds immense promise in revolutionizing the treatment of oral leukoplakia by providing less invasive approaches, yet more efficient and precisely targeted methods.

## Figures and Tables

**Table 1:** Impact of nanomedicines on premalignant lesions.

Beneficial Properties		
Improved internalization into cancer cells	Induction of apoptosis	Bioeffectivenes
Improved drug accumulation at target sites	Enhanced anticancer efficacy	Limited adverse effects
Reduced systemic toxicity	Traversing the tissue with less reduction of blood volume	Customized allocation
Enhanced cytotoxicity of cancer cells	Eliminate dysplastic cells while sparing the healthy ones	

**Table 2:** Advantages and disadvantages of the nanoparticle user in the treatment of oral leukoplakia.

Nanomaterials	Advantages	Disadvantages
Gold nanoparticles	Chemical stability Strong biocompatibility	Au-S bond exhibits limited stability
Silver nanoparticle	Safe delivery carriers	
Iron oxide	Good compatibility Activation of immune cells	Negative contrast effects
Carbon based	Antimicrobial efficacy	Potential to gather in pulmonary tissue
Mesoporous silica based	Consistent porosity Minimal toxicity	
Liposomes	Biocompatibility Minimal immunogenicity	Reduced stability
Micelles	Control liberation of medication	
Dendrimers	Water solubility	
Nanogels	Biodegradability	
Quantum dots	Durability	

**Table 3:** Drawbacks of NPs.

Not suitable for the delivery of proteins and bio-macromolecules
Solid lipid nanoparticles show initial burst drug release
Difficult to handle at times because of particle-particle aggregation
Inorganic NPs like carbon nanotubes have toxic characteristics associated with immune response

**Table 4:** Limitations of conventional therapy.

They possess a non-specific site of action; therefore, it is difficult to differentiate between healthy and tumor cells
Unable to penetrate the biological membranes, as a result of the poor solubility of the drugs
Inability to penetrate into solid tumors, unable to destroy the cancerous cells
They exhibit more systemic cytotoxicity and poor bioavailability
Macrophages engulf the drugs in a short period of time, resulting with a short interaction with cancerous cells
These agents act directly on rapidly growing tumor cells, also damaging the healthy cells resulting in a treatment delay

## Abbreviations

Oral leukoplakia	OL
Photodynamic therapy	PDT
Photosensitizers	PSc
Oral squamous cell carcinoma	OSCC
Reactive oxygen species	ROS
Proliferative verrucous leukoplakia	PVL
Pharmacokinetics-pharmacodynamics	PK/PD
Reticuloendothelial system	RES
Enhanced permeability and retention	EPR
Polyethylene glycol	PEG
Nanomaterials	NMs
Iron-oxide nanoparticles	IONPs
Magnetic resonance imaging	MRI
Magnetic particle imaging	MPI
Ovalbumin	OVA
Carbon nanotubes	CNTs
Multi-walled carbon nanotubes	MWCNTs
Mesoporous silica based nanomaterials	MSNs
Polymeric nanoparticles	PNPs
PEGylated liposomal doxirubicin	PLD
